# Animal Chemistry

**Published:** 1837-04

**Authors:** 


### ANIMAL CHEMISTRY.

*On the Distinctive Characters of Pus, and the Means of recognizing it when mixed with other Fluids, and particularly with the Blood.* By Dr. A. Donné.

This subject is of especial interest at present, on account of attention being again awakened to the reabsorption of pus, and to the mixture of this fluid with the blood, supposed to occur both in phlebitis and metastatic suppurations consequent on traumatic lesions, and in severe internal diseases. Dance, in his excellent memoir on Uterine Phlebitis, says, that "if we reflect on the analogy of the severe symptoms to those produced by the infection of the fluids by miasms, we shall conclude that, if the transport and mixture of pus with the blood during phlebitis are not actually demonstrated, (for direct inspection is often insufficient, and chemical analysis is not of great utility in this enquiry,) yet they are highly probable." In stating the evidence here as merely probable, Dance was right; as in many cases it is impossible to prove the fact, and observers differ in opinion as to the causes of the symptoms. The same may be said of abscesses following wounds, where the alteration of the blood is often admitted from analogy only. In such cases, any method of positively ascertaining the presence of pus in the blood would be highly useful. In the majority of cases where pus has been found mixed with blood, in severe internal diseases, there were centres of suppuration either in the veins or in the parenchymatous and vascular organs, as the liver and lungs. In some fevers true metastatic abscesses have been found, where pus seems to have been deposited without any inflammatory action. M. Piorry inclines towards the opinion that, in these cases, the inflammation of the blood may give rise to the formation of pus in it; but it has not been actually demonstrated that in these diseases there is a mixture of pus with the blood: we do not at present possess any means of proving the presence of pus when it has lost its common physical characters by its mixture with blood.

The first point to determine is, whether pus may be considered as a uniform and identical fluid in all cases; or, at least, whether it always possesses certain essential and characteristic marks, whatever may be its difference of aspect. M. Donné

believes that it does; so does M. Gendrin: an opposite opinion is given by M. Andral, but he insists particularly on the differences in its general appearances. In admitting, with M. Gendrin, that the source of pus is in the blood, that the globules of pus are nothing more than transformed globules of blood, it is not difficult to believe it to be always identical, notwithstanding its varieties: its origin being the same in all cases, it can only differ in its accessory properties, and not in its intimate nature. Whatever kind of pus is examined, it possesses the following properties:

1. Treated with strong liquid ammonia, it is changed into a very tenacious transparent jelly, as has been remarked by many anatomists. This is easily tried in a watch-glass.

2. Examined in the microscope, it appears like a liquid in which a multitude of globular bodies swim, all apparently of the same size, and double that of the globules of blood. The globules of pus are not less than a hundredth of a millimètre in diameter, and the greater number exceed this; whilst the globules of human blood are from 120th to 150th of a millimètre. The appearance of the globules of pus is regular and uniform: they are not lenticular, like those of the blood, but spherical and as if wrinkled: by putrefaction they dissolve, but this requires eight or ten days. Treated by strong liquid ammonia, the globules of pus *do not dissolve*, and are not changed when seen by the microscope. This character is essential.

The property which pus possesses of being changed by ammonia into a tenacious jelly, has not been sufficiently estimated as a test for distinguishing it from all the other animal fluids. Thus, serum of the blood, mucus of the saliva, bile, urine, albumen dissolved in water, when treated with ammonia, do not exhibit the same appearance. A very small quantity of pus, when mixed with a little strong ammonia in a watch-glass, and stirred, gives rise instantaneously, or after a few minutes, to a glairy, thready matter, very similar in appearance to some kinds of expectoration, or to the albumen of an egg. Serum and urine, on the contrary, retain their limpidity; bile loses its viscidity when treated with ammonia.

Mucus is distinguished from pus in the following way:—The expectoration is put in a test-tube with some water, with which it is to be shaken up strongly; and, as M. Gendrin observes, "of all the analyses of pus, the most important, and the one which merits most confidence, is that which consists in treating it with water: the pus precipitates by the cold water, and the yellow pulverulent substance has all the physical characters of pus." But this test may be improved upon by pouring some of the sediment into a watch-glass, and adding the ammonia: if it is pus, the matter is changed into a jelly, and forms a solid mass. If, on the other hand, the expectoration only contains mucus, the liquid remains thready, and is not converted into a glairy and tenacious mass. Semen, to a certain point, may be confounded with pus; for ammonia renders it rather viscid and streaky, but not to the same extent as pus; and, if serum is a little diluted with water or serum, this effect is not produced. But in all cases the microscope renders the distinction easy, as the spermatic animalcules are of a form so characteristic that they cannot be mistaken when once seen. The following case occurred recently:—M. Briquet sent to M. Donné some milky urine from the bladder of a man who died of an encephaloid cancer of the stomach, which had implicated a portion of the bladder: the patient during life had experienced difficulty in micturition. On examining the fluid by the microscope, it was found to contain a large quantity of dead spermatic animalcules. On treating it with ammonia, it deposited a white powdery matter, like semen mixed with water, but neither became thick nor viscid.

If pure milk is treated with ammonia, it acquires no consistence; it only becomes viscid and stringy under certain circumstances; but the milky globules undergo no alteration from the ammonia, and this is a good manner of distinguishing a mixture of milk with blood or serum,—the ammonia instantly dissolving all the globules of blood, and leaving the globules of milk entire. That secretion from the mammæ on the first days after childbirth (called Colostrum,) has a great analogy with pus in the change it undergoes with ammonia: like pus, it becomes a viscid and tenacious mass, which will not break if allowed to fall from one watch-glass into another.

Ammonia acts differently on different parts of the blood: it produces no appreciable effect on the serum, but, if the serum contains a small proportion of pus, it becomes a gelatinous mass; and pus is the only substance capable of producing this effect. This, however, only applies to the serum when separated: when the blood is homogeneous, and no coagulum formed, ammonia acts on it much the same as on pus, forming a viscid jelly, like currant jelly; and it is impossible thus to distinguish blood mixed with pus from pure blood. Its application is therefore confined, as it is not applicable to blood taken from the dead body, where the separation of the solid from the fluid parts of the blood takes place very incompletely: ammonia can consequently only be employed as a test to distinguish pus from other fluids, and not from fluid blood. This is to be done by the microscope.

When recently-drawn blood is examined by the microscope, only globules are seen of a certain well-known form, varying in size with the nature of the animals: those of the human blood vary from 120th to 150th of a millimètre in diameter. Pus is also composed of globules, but their form is so different from those of the blood that it is impossible to confound them. In the first place, the globules of pus are larger than those of the blood; they are hardly ever less than the hundredth of a millimètre in diameter, and generally they occupy a division and a half of a micrometer divided into hundredths of millimètres. Besides this, they differ in form and aspect: the globules of pus have no central nucleus; instead of being bounded by an even and regular edge, they are broken and slightly cut; and in their centre there are small lines crossing one another, and forming a kind of network; they are not flattened, and when, by inclining the object-glass, they are made to circulate, they always appear like spheres. Some of these distinctions are worth considering. Thus, the central nucleus is only seen in fresh blood; in blood obtained from dead bodies at the usual time of examination, it cannot be seen, or, rather, it alone is seen as the coloured envelope of the globule is dissolved. The same may be said of the lenticular form of the globules of blood; for, after some little time, they become spherical. The peculiar appearance of pus, and the greater number of its globules, are the other characteristics which would be sufficient in the greater number of cases, if it did not happen that the most pure and recent blood did not sometimes contain a certain number of globules greater than others, with broken edges, without a central nucleus, composed of irregularly intersecting lines; in fact, very like the globules of pus, but less opaque. These kind of globules are always found in blood beginning to be decomposed, and in the blood taken from dead bodies. M. Donné is aware of only one method of deciding this difficulty, which may be called chemico-microscopic, and is founded on the action of ammonia, which dissolves the globules of blood and leaves untouched those of pus: this can only be seen by the microscope. J. Müller remarked, in the Annals of Physics and Chemistry of Berlin, published in 1832, that "a solution of caustic potash not only removes the coating of colouring matter of the globules of blood, but entirely dissolves their nuclei; and that caustic ammonia acts in a similar way, but with more rapidity." After many trials of this kind of analysis, M. Donné considers the following to be the best mode of operating.

To discover any alteration in the blood, it is at first necessary to examine it with the microscope: for this purpose a drop of blood is to be taken up by means of a glass rod, placed between two very clean slips of glass, and examined with a power which magnifies from two to three hundred times. If there is nothing unusual in the globules, the blood is as pure as we have any means of discovering: this experiment should be repeated several times, by taking specimens from different points of the mass of blood which is to be examined. On the contrary, if other globules are to be seen, the ammonia must be used to determine whether they are globules of pus or partially decomposed globules of blood. If they are only modified globules of blood, the contact of ammonia causes them to disappear instantaneously, so that no appearance of globules can be discovered by the microscope, and nothing else is seen in the liquid placed between the two slips of glass but particles having no particular form, probably consisting of fibrine. If an appreciable quantity of pus is mixed with the blood, there will be no difficulty in distinguishing it with the microscope, as there will not only be some few globules, but many together, forming little compact masses in the midst of the globules of blood; but this alone should not be depended on. A drop of blood is to be placed on a slip of glass, and a very small drop of strong liquid ammonia is to be mixed with it, by making the fluid flow on the glass in different directions; and upon this another slip of glass is to be put: on examining it with the microscope, if there were globules of pus they will remain perfectly distinct, and those of the blood will either be completely dissolved, or a few may escape the action of the ammonia. In the latter case, they will be so pale as to be readily distinguished from the globules of pus: if a long time elapses after the mixture with the ammonia, the pus itself will be found to be dissolved.

The strongest objection to this is, that, if pus cannot by such means be found in the blood, it does not prove that it is not there; for the pus may be in too small a quantity to be readily found, or by accident might not have been placed on the field of the microscope. It is possible also that pus may exist without globules. Finally, we do not know all the changes which take place after death; so that the analysis will be more sure when made on blood collected during life. If, notwithstanding, these experiments will not prove the existence of pus, they will render it very probable.

M. Donné began his experiments by injecting pus into the veins of dogs, from which he took a small quantity of blood at different intervals: in two instances he discovered the pus again, and during many days; but as these experiments were inconvenient and difficult, he collected a little blood as it flowed from a vein in a tube containing a little pus, and shook them up together; the coagulum formed exactly as if the blood was pure, but the serum was slightly bloody: with this mixture he performed the greater number of his experiments. When coagulation had taken place, he could find no globules of pus in the serum, but, on dividing the coagulum, and expressing on a slip of glass a little of the blood it contained, the microscope enabled him to distinguish the globules of pus from those of the blood; and, on adding a drop of ammonia, the former were unaffected, and the latter dissolved. He made several experiments on blood taken from patients where purulent reabsorption was suspected, and says he might cite several where he believed he met with pus mixed with blood. He mentions one instance in which M. Brinet sent him some violet-coloured fluid blood, without saying where it came from, and in which he detected by the previous tests globules of pus. The blood was taken from a person who died from the reabsorption of pus, the consequence of a wound: some of the veins were found to contain pus. But multiplied observations are necessary.

[We have translated this memoir in order that it may attract the attention of medical observers to the microscopic investigations of morbid fluids, and may lead them to try the accuracy of the proposed tests; objects modestly professed by the author. We would say, as a rule, that, unless such statements as are here brought forward are confirmed by more than one investigator, the belief of them should be held very loosely.] *Archiv. Gén. de Méd.* Août, 1836.

